# Retraction Note: lncRNA TUG1 modulates proliferation, apoptosis, invasion, and angiogenesis via targeting miR-29b in trophoblast cells

**DOI:** 10.1186/s40246-022-00389-w

**Published:** 2022-05-18

**Authors:** Qian Li, Jing Zhang, Dong-Mei Su, Li-Na Guan, Wei-Hong Mu, Mei Yu, Xu Ma, Rong-Juan Yang

**Affiliations:** 1 National Human Genetic Resources Center, National Research Institute for Family Planning, Beijing, 100081 People’s Republic of China; 2 Prenatal Diagnosis Center, Shijiazhuang Obstetrics and Gynecology Hospital, Hebei Province, Shijiazhuang, 050011 People’s Republic of China; 3 Genetic Center, National Research Institute for Family Planning, Beijing, 100081 People’s Republic of China; 4 Department of Obstetrics, Shijiazhuang Obstetrics and Gynecology Hospital, Hebei Province, No. 206, East Zhongshan Road, Shijiazhuang, 050011 People’s Republic of China

## Retraction to: Human Genomics (2019) 13:50 10.1186/s40246-019-0237-z

The Editor-in-Chief has retracted this article because Figs. 2 and 5 appear to contain overlapping panels:

- In Fig. 2D, the panels HTR8 si-NC and HTR8 pcDNA3.1 appear to have a considerable region of overlap
- In Fig. 5C, the panels HRT8 si-TUG1 + miR-29b mimic and HTR8 si-TUG1 + miR-29b inhibitor appear to have a considerable region of overlap

The Editor-in-Chief therefore no longer has confidence in the results and conclusions presented.

None of the authors have responded to correspondence from the editor or publisher regarding this retraction.

